# Protection motivation unmasked: Applying protection motivation theory to skepticism toward COVID-19 mask and vaccine mandates

**DOI:** 10.3934/publichealth.2022035

**Published:** 2022-06-10

**Authors:** Robin M. Kowalski, Kenzie Hurley, Nicholas Deas, Sophie Finnell, Kelly Evans, Chelsea Robbins, Andrew Cook, Emily Radovic, Hailey Carroll, Lyndsey Brewer, Gabriela Mochizuki

**Affiliations:** Department of Psychology, Clemson University, Clemson, SC 29634

**Keywords:** protection motivation, vaccine mandate skepticism, mask mandate skepticism, COVID-19, political affiliation

## Abstract

Variants of COVID-19 have sparked controversy regarding mask and/or vaccine mandates in some sectors of the country. Many people hold polarized opinions about such mandates, and it is uncertain what predicts attitudes towards these protective behavior mandates. Through a snow-ball sampling procedure of respondents on social media platforms, this study examined skepticism of 774 respondents toward these mandates as a function of the Protection Motivation Theory (PMT) of health. Hierarchical linear regressions examined Protection Motivation (PM) as a predictor of mask and vaccine mandate skepticism independently and with political party affiliation as a control. PM alone accounted for 76% of the variance in mask mandate skepticism, *p* < 0.001 and 65% in vaccine mandate skepticism, *p* < 0.001. When political affiliation was entered (accounting for 28% of the variance in mask mandate skepticism, *p* < 0.001, and 26% in vaccine mandate skepticism, *p* < 0.001), PM still accounted for significant percentages of variance in both mask (50%) and vaccine (43%) mandate skepticism, *p*s < 0.001. Across regressions, perceived severity, outcome efficaciousness, and self-efficacy each directly accounted for unique variance in mask and vaccine mandate skepticism, *p*s < 0.001; only perceived vulnerability failed to account for unique variance in the regressions, *p*s > 0.05. Specifically, the more severe participants perceived COVID-19 to be and the greater the perceived efficacy of masks and vaccines preventing the spread of COVID-19, the lower participants' skepticism toward mask and vaccine mandates. Similarly, the higher participants' self-efficacy in wearing masks or receiving the vaccine, the lower their skepticism toward mask and vaccine mandates.

## 1. Introduction

At the beginning of the COVID-19 pandemic in the United States, many U.S. employers encouraged their employees to work from home to reduce the spread of the virus [1]. At this time (i.e., prior to vaccine availability), COVID-19 infection rates averaged around 60,000 cases every seven days. After the vaccine was introduced to the public in the spring of 2021, these numbers began to decrease as the public gained immunity to the virus [2]. However, with time, COVID-19 began to mutate into different variants, some of which were more infectious and vaccine-resistant than the original strain [1]–[5]. In response, the CDC released updated guidance in July 2021 which urged the American public to receive COVID-19 vaccinations and engage in preventative measures, as the seven-day moving average of novel COVID-19 cases returned to that of the height of the pandemic in 2020 at 60,000 average daily cases [2]. The continued spread of COVID-19 affected both unvaccinated, in addition to many vaccinated people in the form of breakthrough virus contraction [3]. Evidence shows that fully vaccinated individuals are infected with COVID-19 less often and with less severe symptoms than unvaccinated individuals [1].

Despite this significant increase in COVID-19 cases due to COVID-19 variants, many workplaces throughout the United States required that their employees return to the physical workplace, in some instances strongly encouraging or requiring masks and/or proof of COVID-19 vaccination. President Joe Biden's COVID-19 action plan, for example, requires mask-wearing in “federal buildings, on federal lands, on military bases, and other overseas locations” (i.e., to protect federal workers) [6].

These mandates have become political in nature, resulting in a polarized debate in the United States between Democratic and Republican individuals. A poll of 1729 Americans from the Associated Press Center for Public Affairs Research found that approximately 60 percent of Americans say students and teachers should be required to wear masks while in school [7]. These opinions are disproportionately represented within each political party, as 80 percent of Democrats and 30 percent of Republicans hold that opinion. In that same poll, there were similar political discrepancies regarding school vaccine mandates [8]. However, little is known about what factors or perceptions may lead to such polarization regarding COVID-19 precautions, which has led researchers to apply the attitude change theory known as Protection Motivation Theory (PMT; e.g., [9]–[13]) while researching this issue.

### 1.1. Protection motivation theory and public health crises

At its core, PMT is a social cognition theory developed to understand how people may respond to various health threats, such as seasonal influenza [14], and when considering COVID-19 vaccinations for travel [15]. Developed from expectancy-value theories (e.g., [16]), PMT states that acting in a particular fashion is a function of the expectancy that (1) participating in an act will be followed by some consequence and (2) some value of that consequence [9],[11],[17]. In other words, the propensity to engage in protective behaviors (e.g., receiving a vaccine, wearing a mask) involves the beliefs that people hold regarding the outcomes of engaging (or not engaging) in those behaviors. As originally conceptualized, the PMT included three components: “appraised severity”, “expectancy of exposure”, and “belief in efficacy of coping response” (p. 99) [9]. To this, Maddux and Rogers (1983) [18] added a fourth component, self-efficacy. Previous research has applied the four components of the PMT (perceived severity, perceived vulnerability, outcome efficaciousness of a health behavior in reducing a health threat, and self-efficacy of performing the health behavior) to protective health behaviors such as hand-sanitizing, washing hands, and mask wearing related to reducing the threat of the COVID-19 virus ([19]; see also [20]). In that study, the overall model significantly predicted engagement in protective health behaviors, with perceived severity and outcome efficacy accounting for unique variance.

### 1.2. Skepticism of COVID-19 preventative measures

Beyond PM, other attitudes, such as skepticism, have been examined in relation to engaging in protective behaviors (e.g., [21])^1^. For instance, Jennings et al. examined vaccine hesitancy as a function of government distrust and found that those who indicated they would not receive the COVID-19 vaccine had less trust in the government overall [21]. Similarly, Newport examined how differences in political party ideologies are related to acceptance of and adherence to government mandates related to the pandemic [22]. Specifically, Republicans tended to distrust the government more than Democrats concerning measures to contain the virus.

While demographic variables (e.g., political party; [22]) and beliefs (e.g., trust in government; [21]) have been examined as predictors of skepticism toward mask or vaccine mandates, the literature lacks research concerning PM in relation to skepticism beliefs. Specifically, if an individual has high PM beliefs (e.g., perceived severity, perceived vulnerability), it has yet to be examined if this individual may still hold skeptic attitudes toward vaccine or mask mandates. Given prior research findings [21], however, it is likely that an individual that believes the threat of COVID-19 is severe will be less likely to be skeptical of media portrayals of COVID-19. To further examine this speculation and address the gap in the literature, this paper aims to examine PM in relation to (1) mask mandate skepticism and (2) vaccine mandate skepticism. Additionally, research questions are explored to assess differences in participant views as a function of political party affiliation and vaccination status.

### 1.3. The present study

In summary, the present study advanced two hypotheses regarding the relation of PM to opinions regarding vaccines and mask usage as the primary research question. Secondary aims of the paper were more exploratory in nature including three research questions examining the percentage of variance accounted for in mask and vaccine mandate skepticism by PM with political affiliation included in the equation, reasons for vaccination, and differences in perception between vaccinated and unvaccinated individuals. In particular, this study addresses the following two hypotheses and three research questions:

*Hypothesis 1*: PM will predict levels of vaccine mandate skepticism, such that higher levels of PM will be related to lower levels of vaccine mandate skepticism.

*Hypothesis 2*: PM will predict levels of mask mandate skepticism, such that higher levels of PM will be related to lower levels of mask mandate skepticism.

*Research Question 1*: Will political attitudes account for significant percentages of variance in mask and vaccine mandate skepticism?

*Research Question 2*: How do vaccinated and unvaccinated respondents prioritize reasons for choosing to receive or not receive the vaccine?

*Research Question 3*: Do unvaccinated people express less concern about the COVID-19 virus compared to those who are vaccinated or plan to become vaccinated (e.g., less fear of the virus, lower likelihood of death resulting from the virus) and less perceived efficacy of masks and vaccines?

## 2. Methods

### 2.1. Data screening

Data cleaning consisted of screening for participants via checking for outliers, identification of participants failing data quality checks, and any duplicate responses. Cases of item-level and construct-level missingness in the dataset were deleted if such missingness occurred within the variables of interest. Participants who initiated the survey and failed to complete it were eliminated from the analyses [23].

### 2.2. Participants and procedure

After data screening, 744 individuals (26.7% male, 71.2% female, 1.2% non-binary, 1% other) remained in the sample. Participants completed a Qualtrics survey and were recruited utilizing an online snowballing technique. Participants' ages ranged from 18 to 90 (*M* = 40.68; *SD* = 16.16). Additionally, the sample was predominantly White (95.4%), followed by Asian (3.4%), Black/African American (2.0%), American Indian/Alaska Native (1.1%), and Native Hawaiian/Pacific Islander (0.1%). The Qualtrics survey was distributed via various social media platforms (e.g., Instagram, Facebook, Reddit) and allowed participants to share the survey with their respective networks. Participation involved voluntary completion of the survey questions, which required approximately 20 minutes. The study was approved by the Clemson University Institutional Review Board. Data collection occurred from August to October of 2021, immediately after colleges and universities began implementing mask mandates.

### 2.3. Measures

#### 2.3.1. Demographics and experiences with COVID-19

After providing written consent, participants completed demographic questions pertaining to age, sex, and race, and then responded to a series of questions concerning their experiences with the COVID-19 virus and the COVID-19 vaccine. Specifically, participants were asked if they had been tested for the COVID-19 virus, if they had a positive test result for the virus, and if they had received the COVID-19 vaccine. Response formats for all three questions were dichotomous (*Yes* or *No*) with the exception of the vaccine question, which added a third response option (“I have a plan to receive the vaccine”). The majority of the sample had been tested for the COVID-19 virus (82.4%). Twenty-six percent of the individuals who had been tested for the virus had tested positive. Eighty percent (80.1%) of the sample was vaccinated, 19.4% unvaccinated, with 0.5% planning to get the vaccine. Participants were also provided with a checklist of reasons for receiving or not receiving the vaccine and asked to indicate all that applied. Sample reasons for vaccination included “wanted to protect family members or close friends” and “wanted to serve as an example”. Sample reasons for not getting vaccinated included “concerned about side effects” and “against religious beliefs”.

#### 2.3.2. Mask mandate skepticism

Three items (α = 0.94) measured skeptical attitudes toward mask mandates (adapted from Jennings et al. [21]). Each item asked participants to rate their level of agreement or disagreement with various statements regarding mask mandates: “Mask mandates are necessary for controlling COVID-19” (reverse-scored); “Mask mandates infringe upon my rights”; “Mask usage should be up to an individual not up to an organization.” Each item used the same 5-point Likert-type scale, with 1 being *Strongly Disagree* and 5 being *Strongly Agree*. For each participant, the three item scores were averaged to create a mask mandate skepticism score, with higher values representing higher levels of mask mandate skepticism.

#### 2.3.3. Vaccine mandate skepticism

Three items (α = 0.92) measured skeptical attitudes toward vaccine mandates (adapted from Jennings et al. [21]). Each item asked participants to rate their level of agreement or disagreement with various statements regarding vaccine mandates: “Vaccine mandates are necessary for controlling COVID-19” (reverse-scored); “Vaccine mandates infringe upon my rights”; “Vaccinations should be up to an individual not up to an organization”. Each item used the same 5-point Likert-type scale, with 1 being *Strongly Disagree* and 5 being *Strongly Agree*. These items were averaged to create a vaccine mandate skepticism score, with higher numbers representing higher levels of vaccine mandate skepticism.

#### 2.3.4. Protection motivation

Four items examined each of the components of the PMT. Participants were asked how severe they perceived infection with the COVID-19 virus to be (perceived severity), how likely they thought they were to get the COVID-19 virus (perceived vulnerability), how effective they perceived masks/vaccines to be in preventing the spread of the COVID-19 virus (outcome efficaciousness), and how capable they felt they were of successfully wearing a mask/getting the COVID-19 vaccine to protect against the COVID-19 virus (self-efficacy). All questions were answered on a 5-point Likert-type scale (1 = *Not at All*; 5 = *Extremely*). These items were adapted from the individual items used by Kowalski and Black to assess the ability of the PMT to predict other health behaviors related to COVID-19 [19].

#### 2.3.5. Political affiliation

Political affiliation was assessed through a demographic question. Approximately 50% of the sample indicated they were Democratic (30.1%) or Republican (23.7%), with 22.2% saying they were Independent. The remaining participants reported other (4.6%), none (14.0%), or preferred not to answer (5.4%) and were grouped together with Independents for further analysis. Political affiliation was assessed as a control variable because opinions regarding COVID-19 preventative measures have been shown to vary greatly depending on political affiliation [23]. Based on the categories of Republication, Democrat, and Other (Independent, Other, Prefer Not to Answer), two new dummy-coded variables were created and analyzed. “Other” is reflected as the Constant in the regression equations.

### 2.4. Planned analyses

Two hierarchical linear regression analyses were used to analyze Hypotheses 1 and 2 predicting vaccine and mask mandate skepticism, respectively. Components of PM were entered into the analyses and the variance accounted for by the PM model as well as by each of the PM components individually examined. This analytical approach was selected to allow researchers to determine not only the percentage of variance in outcomes accounted for by the overall PM model, but also the significance of individual model components. Two additional hierarchical linear regression analyses were conducted to test Research Question 1. In the first, mask mandate skepticism was the outcome variable, with political affiliation entered on the first step followed by the components of PM on the second step. An identical regression was conducted predicting vaccine mask skepticism. Reasons for getting vaccinated and for not getting vaccinated were factor analyzed with descriptive information provided about these reasons (Research Question 2). Additionally, analyses of variance (ANOVAs) and multivariate analyses of variance (MANOVAs) by vaccination status were conducted on key study variables (Research Question 3).

## 3. Results

**Table 1.** Dimensions of PMT predicting mask mandate skepticism.

	*B*	SE *b*	*Beta*	*p*
(Constant)	19.15	0.385		0.001
Perceived Severity	−0.937	0.103	−0.212	0.001
Perceived Vulnerability	0.088	0.095	−0.017	0.355
Outcome Efficaciousness	−1.850	0.091	−0.568	0.001
Self-Efficacy	−0.660	0.086	−0.204	0.001

*Note: *R^2^* = 0.76, *p* < 0.001. Definitions of the PM components are provided in the method of the paper.

**Table 2.** Dimensions of PMT predicting vaccine mandate skepticism.

	*B*	SE *b*	*Beta*	*p*
(Constant)	20.40	0.465		0.001
Perceived Severity	−1.161	0.119	−0.259	0.001
Perceived Vulnerability	0.127	0.116	0.025	0.272
Outcome Efficaciousness	−1.251	0.119	−0.378	0.001
Self-Efficacy	0.934	0.109	−0.301	0.001

*Note: *R*^2^ = 0.65, *p* < 0.001. Definitions of the PM components are provided in the method of the paper.

Two linear regression analyses were conducted to determine the PMT's efficacy in predicting mask and vaccine mandate skepticism. In each, components of the PMT [perceived severity, perceived vulnerability, outcome efficaciousness (mask/vaccine), and self-efficacy (mask/vaccine)] were entered as predictors of mask mandate skepticism and vaccine mandate skepticism. The results of the two regressions are presented in Tables 1 and 2. Collectively, the PMT accounted for 76% of the variance in mask mandate skepticism, *F* (4, 725) = 56.5, *p* < 0.001, and 65% of the variance in vaccine mandate skepticism, *F* (4, 723) = 341.67, *p* < 0.001. In each, the only component of the PMT that did not account for unique variance was perceived vulnerability. Importantly, the correlation between mask mandate skepticism and vaccine mandate skepticism was 0.87, *p* < 0.001.

Because of the politically charged nature of attitudes towards masks and vaccines, two identical regressions were run, including political affiliation dummy coded into two new variables on the first step. In the regression predicting mask mandate skepticism, political affiliation accounted for 28% of the variance in mandate skepticism (see Table 3). With this variance accounted for, components of the PMT accounted for an additional 50% of the variance. As seen in Table 3, the conditional means for both Republicans and Democrats differed significantly from Others.

Similar results were obtained in the regression performed with vaccine mandate skepticism as the outcome variable. Political affiliation on Step 1 accounted for 26% of the variance in vaccine mandate skepticism. Again, the conditional means for both Republicans and Democrats differed from Others (Table 4). PM on Step 2 accounted for an additional 43% of the variance. In accordance with Becker, both models with and without political party are provided [24].

**Table 3.** Dimensions of PMT and political affiliation predicting mask mandate skepticism.

		*B*	SE *b*	*Beta*	*p*
Step 1	(Constant)	7.61	0.201		0.001
	Republican	2.59	0.346	0.252	0.001
	Democrat	−3.65	0.319	−0.386	0.001
Step 2	(Constant)	18.00	0.384		0.001
	Republican	1.38	0.193	0.135	0.001
	Democrat	−0.82	0.190	0.190	0.001
	Perceived Severity	−0.87	0.098	−0.197	0.001
	Perceived Vulnerability	0.13	0.090	0.026	0.144
	Outcome Efficaciousness	−1.65	0.090	−0.506	0.001
	Self-Efficacy	−0.65	0.082	−0.199	0.001

*Note: *R*^2^ for Step 1 = 0.28, *p* < 0.001; Δ*R*^2^ for Step 2 = 0.50, *p* < 0.001. “Other” (i.e., participants who were not Democrats or Republicans) is reflected as the Constant in the regression equations. Definitions of the PM components are provided in the method of the paper.

**Table 4.** Dimensions of PMT and political affiliation predicting vaccine mandate skepticism.

		*B*	SE *b*	*Beta*	*p*
Step 1	(Constant)	8.35	0.206		0.001
	Republican	2.49	0.355	0.241	0.001
	Democrat	−3.58	0.328	−0.374	0.001
Step 2	(Constant)	18.91	0.469		0.001
	Republican	1.24	0.234	−0.120	0.001
	Democrat	−1.38	0.226	−0.145	0.001
	Perceived Severity	−1.02	0.114	−0.228	0.001
	Perceived Vulnerability	0.193	0.109	0.037	0.08
	Outcome Efficaciousness	−1.03	0.115	−0.331	0.001
	Self-Efficacy	−0.916	0.103	−0.295	0.001

*Note: *R*^2^ for Step 1 = 0.26, *p* < 0.001; Δ*R*^2^ for Step 2 = 0.43, *p* < 0.001. “Other” (i.e., participants who were not Democrats or Republicans) is reflected as the Constant in the regression equations. Definitions of the PM components are provided in the method of the paper.

**Table 5.** Reasons for getting the COVID-19 vaccine.

Reason	Percentage
Reason	Percentage
Wanted to protect myself from COVID-19	82.0
Wanted to do my part to help control the pandemic	81.5
Wanted to travel and do things I enjoyed before pandemic	62.5
Concerned about possible virus exposures in community	61.1
Wanted to protect a family member or close friend that was high risk for severe disease	58.7
Concerned about possible virus exposures at work/school	55.2
Wanted to serve as an example	51.7
Other	7.0
Employer mandated	3.0

**Table 6.** Reasons for not getting the COVID-19 vaccine.

Reason	Percentage
Concerned about side effects	67.4
Don't need	51.4
Vaccine won't protect me from COVID-19	51.4
Not at risk for serious disease	50.0
Do not believe it's safe	48.6
Do not trust vaccines	43.8
Do not trust the government	39.6
Do not trust the FDA	39.6
Have already had the COVID-19 virus	38.9
Waiting to see how others are affected	31.3
Do not know if it will work	23.6
Other	21.5
My doctor hasn't recommended it	19.4
Believe homeopathic remedies are effective	16.0
Against my religious beliefs	14.6
Other people need it more than I do now	7.6
Don't like	6.0
Not eligible based on medical conditions but would not get it if I could	6.0
Fear of needles	5.6
Do not have time	2.1
Not eligible based on medical conditions but would get it if I could	1.4
Cannot afford	0.0
Concerned about cost	0.0

The reasons participants chose for getting vaccinated or not getting vaccinated are provided in Tables 5 and 6. As shown in Tables 5 and 6, the most common reasons for getting vaccinated focused on “protecting myself” (82.0%) and “wanting to do my part” (81.5%). The most common reasons for not receiving the vaccine included concerns about side effects (67.4%), feeling that they do not need the vaccine (51.4%), and feeling that the vaccine will not protect them (51.4%). Two principal axis factor analyses (direct oblimin rotation) were conducted, one on the reasons vaccinated individuals provided for receiving the vaccine, and one on reasons unvaccinated respondents provided for not receiving the vaccine. The results of these factor analyses are shown in Tables 7 and 8. As shown in Table 7, the factor analysis of the 8 reasons for receiving the vaccine yielded two factors: self-motivation and pandemic control. Self-motivation accounted for 40.80% of the variance (eigenvalue = 3.26); pandemic control accounted for 12.70% of variance (eigenvalue = 1.02). The factor analysis of the 20 reasons for not receiving the vaccine (2 were removed from the analysis because no respondents selected these reasons) yielded 8 factors (see Table 8). These 8 factors with the percentage of variance each accounted for and their eigenvalues were: Self-help (19.29%; 3.86), Dislike of vaccines (9.11%; 1.82), Lack of Need (8.72%; 1.74), Distrust (7.08%; 1.42), Healthcare (6.60%; 1.32), Ethical Considerations (5.68%; 1.14), Consequences of Vaccination (5.21%; 1.04), and Lack of Time (5.03%; 1.01).

**Table 7.** Factor analysis of reasons for receiving the vaccine.

Variable	Factor 1	Factor 2
I was concerned about possible virus exposures in my community	0.791	
I was concerned about possible virus exposures at work or school	0.651	
I wanted to protect myself from COVID-19	0.556	
I wanted to serve as an example to encourage others to take the vaccine	0.531	
I wanted to protect a close family member or close friend that was high risk for severe disease	0.497	
I wanted to travel and do the things I enjoyed before the pandemic	0.412	
I wanted to do my part to help control the pandemic		−0.750

**Table 8.** Factor analysis of reasons for not receiving the vaccine.

Variable	Factors							
	1	2	3	4	5	6	7	8
I believe homeopathic remedies are effective	0.732							
I have a fear of needles		0.947						
I don't like vaccines		0.462						
I believe other people need it more than I do now		0.418	0.362			−0.438		
I do not believe I need the vaccine because I already had COVID-19 infection			0.528					
I do not think I am at risk for serious disease			0.467					
I don't believe I need the vaccine			0.464					
I don't trust the government				−1.031				
I don't trust the FDA				−0.681				
I don't trust COVID-19 vaccines				−0.427				
I am not eligible to get the COVID-19 vaccine based on my medical conditions and I would not if I could					0.713			
My doctor hasn't recommended it					0.514			
I do not believe the vaccine is safe						0.424		
It is against my religious beliefs						0.592		
I am waiting to see how other people are affected by the vaccine							−0.648	
I don't know if a vaccine will work							−0.390	
I am concerned about side effects							−0.377	
I do not have time or cannot miss work to take the vaccine								0.738

*Note: Two items were excluded from the factor analysis due to zero variance (“I am concerned about the cost” and “I cannot afford to pay for the vaccine”). Two other items failed to load in the factor analysis (“I am not eligible to get the COVID-19 vaccine based on my medical conditions and I would get the vaccine if I could”; “I do not think the vaccine will protect me from getting sick with COVID-19”). Factor 1 = Self-help; Factor 2 = Dislike of vaccines; Factor 3 = Lack of Need; Factor 4 = Distrust; Factor 5 = Healthcare; Factor 6 = Ethical Considerations; Factor 7 = Consequences of Vaccination; Factor 8 = Lack of Time.

**Table 9.** Comparison of vaccinated versus unvaccinated respondents.

Variable	Unvaccinated	Vaccinated	Plan to Receive Vaccine	*F*	*p*	η^2^
Been tested for virus	74.8%	84.2%	75%	3.61	0.03	0.01
Tested positive for virus	42%	16.7%	25%	34.67	0.001	0.10
Fear of COVID-19 virus	1.76_a_	3.48_a_	2.75	130.12	0.001	0.264
Likelihood of death from virus	2.32_a_	3.38_ab_	2.00_b_	58.66	0.001	0.139
Perceived severity of virus	2.65_ab_	3.84_a_	3.50_b_	99.38	0.001	0.215
Perceived vulnerability	2.61	3.55	3.25	3.95	0.02	0.011
Outcome efficacy of vaccine	1.82_ab_	4.24_a_	3.50_b_	376.76	0.001	0.510
Self-efficacy of getting vaccine	1.72_ab_	4.81_ac_	3.50_bc_	987.42	0.001	0.732
Outcome efficacy of masks	1.77_a_	3.71_ab_	2.50_b_	180.28	0.001	0.332
Self-efficacy of masks	2.57_a_	4.45_a_	3.50	163.75	0.001	0.311

*Note: Means or percentages in a single row that share a common subscript differ significantly, *p* < 0.05. Degrees of freedom for first two rows = 2740); for all other variables: *df* = 2724.

Additionally, Table 9 presents a comparison of individuals who reported their status as vaccinated, unvaccinated, or plan to receive the vaccine along key variables in the study. Analyses of variance (ANOVA) by vaccination status (vaccinated/unvaccinated/plan to get vaccine) were conducted on the two variables assessing whether participants had been tested for the COVID-19 virus and, among those, whether they had tested positive. A multivariate analysis of variance (MANOVA) was conducted on the remaining key variables included in Table 9. Following the significant multivariate effect of vaccination status, *F* (16, 1436) = 54.40, *p* < 0.001, η^2^ = 0.76, univariate ANOVAs were examined. Compared to unvaccinated respondents, vaccinated individuals reported more fear of the COVID-19 virus and a greater perceived likelihood of dying from the virus. Those who were vaccinated also perceived the virus to be more severe, perceived both masks and vaccines to be efficacious in protecting against the virus, and thought of themselves as capable of wearing a mask and getting the vaccine. Despite overall significance, post hoc tests revealed no significant differences between groups on the variables examining whether participants had been tested for the virus, whether they had tested positive for the virus, and perceived vulnerability to the virus, *p*s > 0.05. The likely explanation for the failure to find between group differences is the very low variance for these items. Data are available at https://osf.io/rjcpf/?view_only=58579e3ac63a45cca47fab9d2c251809.

## 4. Discussion

Based on the results of the current study, the PMT provides a useful theoretical model for examining attitudes toward mask and vaccine mandates. Overall, the PMT accounted for substantial and significant percentages of variance in both mask and vaccine mandate skepticism, supporting Hypotheses 1 and 2. All components of the PMT uniquely predicted mask and vaccine mandate skepticism except for perceived vulnerability. The more severe participants perceived COVID-19 to be and the greater the perceived efficacy of masks and vaccines preventing the spread of COVID-19, the lower participants' skepticism toward mask and vaccine mandates. Similarly, the higher participants' self-efficacy in wearing masks or receiving the vaccine, the lower their skepticism toward mask and vaccine mandates. Due to the continued exposure that everyone has to information about COVID-19 in the media, with a particular focus on the number of cases locally and nationally, it is likely that everyone perceives themselves to be vulnerable to getting COVID-19 at some point. Indeed, only 3% of the sample indicated that they were not at all likely to get the virus.

Notably, the PMT continued to account for a significant percentage of variance in mask/vaccine mandate skepticism after entering political affiliation (dummy coded) on Step 1. Because political affiliation accounted for substantial and significant portions of variance in mask and vaccine mandate skepticism, Research Question 1 was supported, highlighting the politicized nature of issues surrounding COVID-19, mask-wearing, and, in particular, vaccines. This is reinforced by the finding that, among participants who had not received a vaccine, 39.6% did not trust the government. This finding mirrors Jennings et al.'s study [21] regarding lack of governmental trust predicting mistrust in COVID-19 vaccines. Additionally, the issue continues to permeate itself in the realm of politics. Our findings support those of Newport [22], such that differences in political party affiliation account for significant variance in attitudes toward mask or vaccine mandates. A possible explanation for this finding, as Newport stated, is that differences in political party affiliation may also result in differences in the communication forms used to inform individuals about the COVID-19 virus [22]. In other words, some news channels may depict information more accurately than others. This difference in communication may lead to differing levels of trust for the government and the information being shared regarding COVID-19.

Research Question 2 addressed the reasons why people choose or refuse vaccination. While possible reasons were provided to participants, the study was primarily concerned with the percentage of individuals who endorsed each option. Among vaccinated participants, the most frequently endorsed reasons for getting the vaccine were wanting to protect themselves and do their part to help control the pandemic. Except for the 3% of vaccinated individuals who received vaccines because of employer mandates, at least 50% of vaccinated individuals thought all of the other reasons were important. Among unvaccinated individuals, the greatest percentage were concerned about side effects, felt they did not need the vaccine, or felt it would not protect them against the COVID-19 virus. Results of the factor analysis conducted on reasons for receiving the vaccine indicated seemingly clear self-motivated considerations. Reasons for not getting the vaccine were much more dispersed, with many of the factors indicative of external motivators (e.g., distrust, consequences, and dislike of vaccines).

The relative importance applied to the different reasons should factor into health communications regarding mask and vaccine efficacy. Disseminating information about vaccines, for example, that carries little weight in influencing people's decisions to get the vaccine is not effective. Alternatively, communicating accurate information about side effects and the consequences of not receiving the vaccine may be met with greater compliance with recommended guidance. There appeared to be more variability in the reasons unvaccinated individuals had not yet received the vaccine. While this could simply be the result of this group having a longer list of reasons to choose from on the survey, the factor analysis suggests that motivations for not getting the vaccine are, indeed, more disparate among the unvaccinated.

Research Question 3 raised the question of whether people who are unvaccinated express fewer concerns about the COVID-19 virus than those who are vaccinated or plan to become vaccinated (e.g., less fear of the virus, less likelihood of death resulting from the virus, and less perceived efficacy of masks and vaccines). The results of the current study suggest that they do. Compared to vaccinated respondents, unvaccinated individuals expressed significantly less fear of the COVID-19 virus and less likelihood that they would die from the virus. Compared to vaccinated individuals, unvaccinated respondents also perceived the COVID-19 virus to be less severe, thought that both masks and vaccines were less efficacious in stopping the spread of the COVID-19 virus, and perceived themselves to have less efficacy in wearing the masks or getting the vaccines. These findings are essential regarding public health communications designed to increase people's willingness to get the vaccine and which areas need to be targeted. Increasing awareness about the COVID-19 virus through campaigns is not likely to increase participation in getting the vaccine. However, increasing perceptions of the efficacy of the vaccine or the severity of the virus as well as continuing to send the message that cases among unvaccinated individuals are more severe and more likely to result in death than those among vaccinated individuals (e.g., [3]) may result in greater willingness to receive the vaccine and wear masks.

## 5. Limitations

Several limitations exist in the study. First, the study relied on surveys as the only method of data collection. Given the sensitive nature of the topic under investigation, this method afforded the researchers information that interviews may not have. Second, the current study used single item assessments of the four PM components. However, previous research has shown that these single-item measures correlate significantly (*p* < 0.01) with multi-item assessments of the same constructs, leading us to have confidence in the psychometrics of the measures [19]. Third, the demographics of the study were predominantly white and female. Thus, it is possible that the results of the study may lack generalizability to the greater population. However, the results obtained from this study examining the utility of the PMT in predicting mask and vaccine skepticism are similar to those of other studies examining people's willingness to engage in positive health behaviors designed to discourage the spread of the COVID-19 virus (e.g., [19]). Fourth, because no incentives were offered to participants, it is possible that those who responded were simply more polarized in their opinions, including their political opinions, and more willing to share those opinions. However, based on the data at hand, we have no reason to think that there was a polarization effect. Fifth, the current study examined skepticism toward mask and vaccine mandates rather than attitudes toward masks and vaccines as means of preventing the spread of COVID-19 or reducing the severity of infection with the virus. Previous research [19] has examined the efficacy of the PMT in predicting people's attitudes toward masks and other protective health behaviors, such as social distancing. It is certainly possible that someone could have positive attitudes toward the use of masks and vaccines yet be against mask and vaccine mandates. Finally, regarding reasons for vaccination or not being vaccinated, the list of reasons for being unvaccinated was longer than the list of reasons for being vaccinated. Thus, this length difference may have caused range restriction as less response options were available for vaccinated individuals, which can explain why reasons for vaccinated individuals had less variability than unvaccinated individuals.

## 6. Conclusions

This study contributes to the literature by examining PM in relation to attitudes toward mask and vaccine mandates. Specifically, PM significantly predicted levels of mask and vaccine mandate skepticism beyond the differences explained by political party affiliation. While prior research has examined PMT in relation to protective behaviors for other public health crises (e.g., seasonal influenza; [18]), this study is the first to examine PMT regarding attitudes toward mandates of protective behaviors.

Overall, results demonstrate the influence that political party affiliation, as well as PM, has on an individual's likelihood of accepting mask or vaccine mandates. Especially among those who plan to be vaccinated, it is essential to communicate correct and relevant information pertaining to health crises such as COVID-19. This notion is also supported by the amount of mask and vaccine mandate skepticism that was attributed to political party affiliation as certain media sources may depict the virus differently. As COVID-19 continues to shape the climate of the United States, educating the public of correct information sources should continue to be a top priority for researchers. Given the politicized nature of these debates, politicians, regardless of political party, should include information related to COVID-19 severity and vaccine efficacy in their messages to constituents. It is likely that discrepancies in the form of communication about the virus influence an individual's likelihood of engaging in protective behaviors, which is an influence that could ultimately mean the difference between life and death.

## Footnotes

1 It is important to note that mandate skepticism is not subsumed within the outcome expectancy component of the PMT. The extent to which we perceive that a behavior may be efficacious in warding off a health threat is not the same thing as thinking that a preventive health behavior infringes upon one's rights.
